# The Healthy School Canteen Programme: A Promising Intervention to Make the School Food Environment Healthier

**DOI:** 10.1155/2012/415746

**Published:** 2012-05-28

**Authors:** Fréderike Mensink, Saskia Antoinette Schwinghammer, Astrid Smeets

**Affiliations:** The Netherlands Nutrition Centre, P.O. Box 85700, 2508 CK The Hague, The Netherlands

## Abstract

The environment can exert a strong influence on people's food decisions. In order to facilitate students to make more healthy food choices and to develop healthy eating habits, it is important that the school food environment is healthy. The Healthy School Canteen programme of The Netherlands Nutrition Centre is an intervention that helps schools to make their cafeteria's offering healthier. A descriptive study was conducted by an independent research agency to survey the perceptions, experiences, and opinions of users of the programme (school directors, parents, students, and health professionals). Results show that directors and students of participating schools perceive their cafeteria's offering to be healthier after implementing the programme than prior to implementation. Next, further important results of the study are highlighted and relations with other projects, caveats, and practical recommendations are discussed. It is concluded that the Healthy School Canteen programme is a promising intervention to change the school food environment but that further research is needed to ultimately establish its effectiveness. Also, it will be a challenge to motivate all schools to enroll in the programme in order to achieve the goal of the Dutch Government of all Dutch school cafeterias being healthy by 2015.

## 1. Introduction

With 14% of young people in The Netherlands being overweight [1], the prevalence of overweight continues to grow, and many teenagers have an unhealthy food pattern containing too much saturated fats, sugars, and a lack of dietary fibre [2]. The fact that children spend many hours at school each day, including lunchtime, causes the school environment to be an important out of home setting where children consume at least one main meal a day. Almost 90% of all secondary schools in The Netherlands have a school cafeteria and/or soft drink vending machines, and 80% have vending machines selling snacks and candy bars [3]. With one in three schools selling pizza and one in five selling deep-fried products, almost half of all schools selling candy bars and a lack of fresh fruit in 57% of the schools [3], there is still a lot to improve when it comes to offering healthy foods in the school cafeteria. In this paper, we will first elaborate on why it is important to offer healthy food in school cafeterias and then introduce the Healthy School Canteen programme, an intervention that is aimed at making the school food environment healthier. In the remainder of this paper, we will discuss a descriptive study that was conducted to assess perceptions and opinions of parties that (have) participate(d) in the programme.

The idea that environmental factors can be important in shaping human behaviour is not new. In the 1930s, Lewin already emphasizes in his field theory that both the person and the environment need to be taken into consideration when studying human behaviour [4]. Lewin, considered to be the founding father of social psychology, conventionalised human behaviour as a function of both the person and the environment. This idea became known as Lewin's equation: *B* = *f*(*P*, *E*). From this heuristic, it follows that behaviour is the result of an interplay of one's personal characteristics and the situation (that contains both physical and social elements) in which the person operates. This perspective provides a useful starting point from which to consider eating behaviour. Specifically, it could help to explain that good and strong intentions to eat healthily (person factors) are most of the time not enough to prevent people from making unhealthy food choices. Rather, temptations that lurk in one's direct environment such as the smell of hamburgers or seeing friends eating candy bars can be very powerful in shaping people's actual eating behaviour.

Although Lewin's equation was quite revolutionary in his days and sparked some debate among fellow scientists, his conceptualization is widely acknowledged nowadays. Also, in the domain of eating behaviour, there is ample evidence now that environmental cues can influence people's eating decisions, both consciously and unconsciously. For example, research has demonstrated that the way food is presented, portioned, and packaged in one's direct environment can affect the amount of food that one consumes. Specifically, larger serving portions and packages usually allure people to consume more food, which in turn leads to greater energy intake [5, 6]. In addition, the accessibility and presentation of foods can influence people's food choices in such a way that the more accessible or easy to reach certain types of food are, the more they are being consumed [7, 8].

Interestingly, the impact of the environment on people's food choices and eating behaviour is dependent on the way in which people make their food decisions. Specifically, decision-making and choice behaviour usually results from one of two distinct cognitive processes: reflective or impulsive processes [9, 10]. When decision-making is powered by the reflective system, people think carefully and rationally and they usually act upon their intentions. On the other hand, when people operate through the impulsive system, they act more automatically and spontaneously and are usually led by impulses. It is under these circumstances that environmental cues can strongly affect people's decisions and behaviour [9]. When we apply these insights to the current topic of eating behaviour, it is to be expected that the environment strongly influences such behaviour when people make food choices via the impulsive system (as opposed to the reflective system). Research on habits and information processing has demonstrated that when behaviour has become habitual and when people are not motivated or cognitively involved enough (or are too distracted) to engage in effortful reasoning and deliberation, their decision making will likely be powered by the impulsive system [11, 12]. More specifically, this implies that when people have well-developed eating habits and are not very much involved in their food choices (and as a result do not invest much time and effort in thinking about their choices), the environment is likely to determine their eating behaviour to a large extent.

A recent study indicates that most students do not consider their eating patterns important and making healthy food choices is not a top-of-mind issue for them [13]. Also, meals and foods are consumed during breaks, which are for most students social events in which they communicate and hang out with each other. These two facts imply that students, when they are at school deciding what to eat for lunch, will most probably not be motivated enough or too distracted to engage in deliberate decision making about their eating behaviour. Therefore, it is very likely that most students' choices about what to eat are largely based on decision making through the impulsive system. As a result, environmental cues, such as the mere presence of unhealthy food items, portion and packaging sizes, and tempting smells or displays of unhealthy food, will most probably have an impact on students' eating behaviour. In line with this, students themselves also indicate that they are influenced by the presence of unhealthy food in the school cafeteria. More specifically, they admit to be tempted when they see or smell palatable and unhealthy food [14]. For this reason, many Dutch students indicate that in their opinion schools should only sell healthy products [14]. Still, the majority of school cafeterias offer a large amount of unhealthy food products, and the school environment contributes in this way to the development of unhealthy eating patterns in young people.

At the same time, school cafeterias offer great potential to improve students' eating behaviour. When taken into consideration that most students tend to engage in impulsive decision making, when it comes to their food, this implies that environmental cues can also “nudge” them in the direction of more healthy choices. When cafeteria offerings would be predominantly healthy and healthy food would be made more attractive (e.g., appealing presentation, putting it on display), it is to be expected that this would increase healthy choices. And indeed, a study by TNO has demonstrated that this can be a fruitful and effective means of encouraging healthy eating behaviour in students: changing the offering of vending machines into low-calorie candy, snacks, and soft drinks, resulted in students choosing these healthy products more often [15]. As a result, they had a lower calorie intake than students of schools with vending machines in which products with a lot of sugar and fat prevailed.

 Another reason why targeting students offers great potential to improve healthy eating habits is that eating habits that are formed early in life may persist into adulthood [16] and that, once an unhealthy habit has been established it is difficult to change [17]. Therefore, promoting and establishing healthy habits in young people is probably more effective and fruitful than trying to change unhealthy habits later in life. In addition, schools are increasingly indicated as key settings for interventions related to healthy eating. Health promotion in schools is worth the effort, because it can contribute to healthier behaviour in pupils, higher academic achievements, and a reduction in school drop-out levels [18]. At the same time, the school setting is an important context for health promotion because it reaches a large proportion of the population for many years [19]. It also offers a safe environment to practice new skills [20]. These skills have an effect on the possibility of young people to protect themselves against health risks and can positively affect their lifestyle into adulthood [20]. In sum, interventions aimed at changing students' eating behaviour in the school setting have a lot of potential.

Given the influence the environment can exert on students' food choices, it is crucial to create a healthy food environment in schools that facilitates students to choose healthy food products. In this way, students are enabled to develop healthy eating habits from which they can benefit the rest of their lives. With this particular aim the Healthy School Canteen programme was developed. The Healthy School Canteen programme of The Netherlands Nutrition Centre is an environmental intervention designed to create a healthy food environment and promote healthy food choices in secondary schools and schools for vocational training in The Netherlands. This intervention entails a multicomponent strategy involving all parties: students, teachers, parents, school boards, canteen employees, Municipal Health Services, and caterers.

The programme consists of a four-step roadmap for school working groups, consisting of (1) an Inventory (what is the current state of affairs regarding cafeteria offerings, curriculum and policy?), (2) an Action Plan (setting goals and corresponding actions), (3) an Implementation Phase (implementing the action plan), and (4) an Evaluation (what has been achieved?). While completing these four steps, the school is guided towards a healthy school canteen in their own tempo. As health promoting interventions are more effective when they are structurally implemented in schools and the set up is comprehensive [18], the Healthy School Canteen programme not only motivates schools to change the offerings in the school cafeteria but also encourages them to embed knowledge of healthy nutrition in the curriculum and to develop healthy school food policies. Municipal Health Services play an important role in guiding schools through the process of change. As we have learned from experience, not every Municipal Health Service has enough time and manpower to support all schools in need of support in their region. To be able to support the schools in need, an important additional component of The Healthy School Canteen programme was created: the “Canteen Brigade.” This brigade consists of dieticians employed by The Netherlands Nutrition Centre, who give tailored advice to schools and, if necessary, visit schools to provide tailored advice and support on site.

Since the pilot study in 2002, almost one third of all secondary schools in The Netherlands have worked with the programme [3]. In 2006-2007, 11% of all secondary schools participated; in 2010-2011, 29% indicated to have participated in the programme in the last four years [3], which is a substantial increase. To motivate schools to enroll in the programme, a Healthy School Canteen Stimulation Award competition has been organized biannually since 2006. This competition challenges schools to submit an action plan that describes the steps they will take to create a healthier food offering. After 6 months, a report must be handed in, in which the achieved goals are described. The school that has accomplished the most structural changes will win the award.

In 2010, a descriptive study among users of the programme was carried out by an independent research agency to survey the perceptions, experiences, and opinions of school directors, parents, students, and health professionals with the programme [21]. This study was undertaken to gain more insight on perceptions of users of the programme and to define factors that could help to improve the programme. In this descriptive study, the following issues were addressed: (1) perceptions of the school's cafeteria offerings, (2) the way in which the school's cafeteria was managed, (3) the role of the Municipal Health Service, (4) continuation of the programme, (5) additional value of the Stimulation Award competition, (6) parents' involvement in the cafeteria's offering, and (7) possible factors that stimulate nonparticipating schools to enroll in the programme. (The Canteen Brigade has been active since the end of 2009 and, for this reason, was not part of the research study. Schools for vocational training started participating in the programme from 2011 and, for this reason not part of the research study.). In the remainder of this paper, we will elaborate on this research and present and discuss the most important results.

## 2. Method

### 2.1. Recruitment and Procedure

Contacts of schools that participated in the Stimulation Award competition in 2006-2007, 2008-2009, and/or 2009-2010 were approached by e-mail to provide us with the e-mail address of their school director, student council, and parent council. A dataset with e-mail addresses of every Dutch secondary school was used to invite nonparticipating schools to participate in this study.

Subsequently, directors of participating schools (school directors of schools that (have) participate(d) in the Healthy School Canteen Stimulation Award competition at one point in time during the years 2006–2010) and nonparticipating schools and parents and students of participating schools, were invited to participate in the study and were sent links to online questionnaires. In total, four online questionnaires were sent out; one to school directors of participating schools, one to parent councils of participating schools, one to student councils of participating schools, and one to school directors of non-participating schools (respondents of participating schools only had to answer questions that were relevant to them; respondents of schools participating in the school year 2009-2010 for instance did not have to answer questions about continuation of the programme as they had just started). Questionnaires were sent to 153 directors of participating schools, 139 parent councils, 137 student councils, and 708 nonparticipating schools.

In addition, interviews were held with ten school directors of participating schools of the Stimulation Award competition in the school year 2006-2007 and 2008-2009, who were randomly selected and approached by telephone and e-mail with the request to participate (school directors of schools participating in the Stimulation Award competition at that specific time were not approached to participate, because questions about continuation of the programme would not be relevant yet). All interviews were conducted by an independent research agency. Finally, an expert meeting was held with 12 health promoters of involved Municipal Health Services to discuss their experiences supporting schools during the process of changing the offering of their school cafeteria by implementing the Healthy School Canteen programme. For this expert meeting, all contacts of 28 Municipal Health Services were invited by e-mail to participate. The expert meeting was conducted by an independent strategy development agency.

### 2.2. Questionnaires

**Directors**

The online questionnaire for school directors of participating schools first assessed their perception of the school's cafeteria offerings. Specifically, the following questions were asked: “How was the ratio healthy/unhealthy offerings in the school cafeteria before start of the programme?” and “How is the ratio healthy/unhealthy offerings in the school cafeteria at this moment?”. Answers were given on 5-point Likert-type scales (1 = *almost entirely unhealthy products*, 5 = *almost entirely healthy products*).

Subsequently, questions about the programme, the degree of external support and continuation of the programme, and the Stimulation Award competition were presented. Specifically, the following questions were asked

- “Who manages the school cafeteria?” with three response options; (1) internal management, (2) external management (professionally organized catering), or (3) otherwise, namely….
- “Did your school receive support from the Municipal Health Service?” with two response options; “yes” or “no.”
- “How do you evaluate the support given by the Municipal Health Service?” Answers were given on 5-point Likert-type scales (1 = *quite insufficient*, 5 = *very good*).
- “Which continuation activities did your school carry out?” with response options like “structural change in canteen offerings” and “development of a school food policy”.
- “The Healthy School Canteen Stimulation Award competition motivated to enroll in the Healthy School Canteen programme.” Answers were given on 5-point Likert-type scales (1 = *totally disagree*, 5 = *totally agree*).

**Students**

The online questionnaire for student councils of participating schools also first assessed their perception of the school's cafeteria offerings. The student councils were asked to represent the opinion of all students when answering the questions.

Specifically, the following questions were asked: “How was the ratio healthy/unhealthy offerings in the school cafeteria before start of the programme?” and “How is the ratio healthy/unhealthy offerings in the school cafeteria at this moment?”. Answers were given on 5-point Likert-type scales (1 = *almost entirely unhealthy products*, 5 = *almost entirely healthy products*).

Subsequently, questions were asked about involvement and perception of the students regarding the programme. Specifically, the following question was asked: “Were students involved at the start of the programme?”. There were three response options; (1) yes, (2) no, or (3) I do not know. In addition, students were asked to respond to the statement: “Our students acknowledge the importance of the Healthy School Canteen programme”. Answers were given on 5-point Likert-type scales (1 = *totally disagree*, 5 = *totally agree*).

**Parents**

The online questionnaire for parent councils of participating schools explored involvement and perception of the parents regarding the programme. The parent councils were asked to represent the opinion of all parents when answering the questions.

Specifically, the following question was asked: “Were parents involved/informed at the start of the programme?”. There were three response options; (1) yes, (2) no, or (3) I do not know. Also, the following statement was used: “Parents have a say in selection of school canteen offerings”. There were three response options; (1) yes, (2) yes, but only through the parent council, or (3) no. Next, parents were asked to respond to the following statements: “Parents know what is offered in the school cafeteria” and “Parents have a say in the cafeteria's offering”. There were three response options; (1) yes, (2) no, or (3) I do not know. Finally, parents were asked to respond to the statement: “Parents acknowledge the importance of the Healthy School Canteen programme.” Answers were given on 5-point Likert-type scales (1 = *totally disagree*, 5 = *totally agree*).

**Nonparticipating Schools**

The online questionnaire for school directors of nonparticipating schools assessed their perceptions of the school's cafeteria offerings. Specifically, the following question was asked: “How is the ratio healthy/unhealthy offerings in the school cafeteria at this moment?” Answers were given on 5-point Likert-type scales (1 = *almost entirely unhealthy products*, 5 = *almost entirely healthy products*). Also, statements were used to determine which factors would motivate them to participate in the Healthy School Canteen programme. Specifically, directors were asked to respond to the following statements: “The required time investment has to be met for by the school” and “The required finances have to be met for by the school.” Answers were given on 5-point Likert-type scales (1 = *totally disagree*, 5 = *totally agree*).

### 2.3. Interviews with Directors

The interview design was based on the online questionnaire and consisted of in-depth and additional questions about the school director's participation, support during execution of the programme, continuation, Stimulation Award participation, and possible improvements of the programme.

### 2.4. Expert Meetings

**Health Professionals**

The aim of this meeting was to obtain more insight in their experiences, needs and the role Municipal Health Services play within the programme. Participants were asked to indicate which components of the programme should be continued, which components should be eliminated and with which components the programme should be enriched. More specifically, one of the statements that was used was “Municipal Health Services perceive the Stimulation Award competition to be an incentive for schools.”

## 3. Results

### 3.1. Questionnaires

Response rates were as follows: 62,7% (*n* = 96) of school directors of participating schools filled in the questionnaire, 54% (*n* = 75) of the parent councils, 38,7% (*n* = 53) of the student councils, and 25,6% (*n* = 181) of nonparticipating schools. Of the 181 nonparticipating schools, 39 were eliminated from the study because they were already implementing the Healthy School Canteen programme (*n* = 29) or were interested in doing so (*n* = 10). Hundred and thirty-five schools were not motivated to enroll in the programme in the nearby future. These schools received the questionnaire. The most important findings will be discussed below.

**Perceived Cafeteria Offering**

Differences in mean scores of participants on the questions regarding the offering before and after implementing the programme (at this moment) were compared with paired *t*-tests. Differences in mean scores of school directors of participating and nonparticipating schools on the question regarding the offering “at this moment” (for participating schools, this was after implementing the programme) were compared with an independent *t*-test.

Analyses showed that both school directors and student councils perceived the offering before and after implementation to be significantly different. Specifically, directors perceived the cafeteria offering to have shifted from relatively more unhealthy products before start of the programme (*M* = 2.17) to more healthy products at the present moment (*M* = 3.76; *M*-change = −1.60; SD = 1.12), *t*(83) = −13.05, *P* < 0.0001. For students, a similar pattern emerged: they also perceived the cafeteria offering to have shifted from more unhealthy products before start of the programme (*M* = 2.55) to more healthy products at the present moment (*M* = 3.80; *M*-change = −1.25; SD = 1.62), *t*(39) = −5.28, *P* < 0.0001. In contrast, directors of nonparticipating schools perceived (at this moment) that the number of unhealthy and healthy products being offered at their cafeteria was equal (*M* = 3.06). Analyses showed there appears to be a significant difference in perceived offering “at this moment” between school directors of participating schools and school directors of nonparticipating schools (SD = 1.39), *t*(217) = 4.68, *P* < 0.0001.

**Cafeteria Managing**

69% of schools indicated the school cafeteria had internal management, and 31% of schools indicated the school cafeteria had external management. In addition, there appeared to be a relationship between whether the cafeteria was catered by the school or an external party: 32,7% of schools with a cafeteria managed by the school itself against 12,0% of schools with an external caterer indicated to have an almost completely healthy offering in the school cafeteria (Spearman's *r* = .26, *P* = 0.015). Apparently, cafeterias that are managed by a professionally organised external caterer have a less healthy offering than cafeterias managed by the school.

**Role of Municipal Health Service**

73% of participating schools indicated they were supported by a Municipal Health Service, and the majority are (very) content with this support: sufficient (32%), good (42%), and very good (17%). Only schools that started the programme during the school years 2006-2007 and/or 2008-2009 were questioned about continuation of the programme (*n* = 48).

**Continuation of Programme**

Three activities that have often been undertaken as continuation of the programme are structural changing the food on offer in the cafeteria (69,2%), making healthy eating a part of the regular curriculum (64,1%), and changing the school food policy (61,5%).

**Additional Value Stimulation Award**

A majority (75,3%) of directors of participating schools (completely) agreed that the Stimulation Award competition motivated to enroll in the healthy School Canteen programme.

**Parents' Involvement with Programme and Cafeteria Offering**

59,2% of the students and 47,9% of the parents say they were involved at the start of the programme. Of the parents, 15,2% reported they had a say on what is being sold in the cafeteria, and almost half of the parents indicated they know what is being sold (48,3%).

**Importance of Programme**

55,3% of the students and 80% of the parents (totally) agreed with the statement that the Healthy School Canteen programme is important.

**Stimulating Factors for Nonparticipating Schools**

Important factors for nonparticipating schools to start with the program are time and finances: 78,5% indicated enough time is (very) important, and 73,3% pointed to sufficient finances as being (very) important.

### 3.2. Interviews

All 10 interviewed school directors were positive about the Stimulation Award competition and find it a good initiative. The interviews also revealed that schools are in need of “role model schools” and experiences of other schools for inspiration. All 10 interviewed school directors indicated support of students, teachers, and parents was created and that support of the school director was sufficient. Seven school directors indicated the school was supported during the programme by the Municipal Health Service, and 5 school directors indicated they received support from The Netherlands Nutrition Centre. All ten interviewed school directors indicated healthy food had been included in the school food policy.

### 3.3. Expert Meetings

During the meeting with Municipal Health Services, 12 professionals were present. Below the most important insights are discussed.

 Municipal Health Services feel that the Stimulation Award competition is a relevant part of the Healthy School Canteen programme, and according to them, the programme is a good way to highlight the importance of healthy nutrition in secondary schools. However, they indicate that there should be more time to execute the programme when participating in the Stimulation Award competition, partly because they need enough time to recruit schools.

## 4. Discussion

It appears that schools participating in the Healthy School Canteen programme have been successful in creating improvements in their school cafeteria offerings due to implementing the programme, as they report to have healthier offerings compared to nonparticipating schools. Also, the Stimulation Award competition is seen as a motivator to enroll in the programme both by schools and Municipal Health Services. It is encouraging that both school management and students perceive that their cafeteria's offering has positively shifted into the direction of more healthy than unhealthy food products. A recent study on overweight prevention in secondary schools in The Netherlands in 2010-2011 supports this finding and states that, for a large number of schools, improvements in their cafeteria were realized (at least in part) by participating in the Healthy School Canteen programme [3]. The overall picture indicates that the school cafeteria offerings in The Netherlands have become healthier compared to 2006-2007. This is probably largely due to an increase in healthy products being offered and a decrease in unhealthy products [3].

The Healthy School Canteen programme and the present findings are also relevant in light of the overall aim of the Dutch government to realize healthy school canteens in all schools in The Netherlands by 2015. The present findings provide a first indication that the Healthy School Canteen programme could be a powerful contributor in achieving this goal. This goal was also adopted by the Dutch Covenant on Healthy Weight. This Covenant is a collaboration of a total of 27 actors from (national and local) governments, industry and civil society organizations, which are collectively committed to fight against the rising trend of overweight and obesity. The main goals of the Covenant are increasing awareness of health risks related to overweight and obesity and achieving an arrest in the evolution of overweight and obesity in children and adults. Within this Covenant a Manifest on Healthier Food in schools was realised in 2011 in which parties agree to work together towards schools where the food offered in the school cafeteria is healthy to a minimum of 75% according to the Dutch guidelines for healthy food.

### 4.1. Relations with Other Initiatives

Across Europe, many countries take action to positively change the school food environment. For example, English chef Jamie Oliver has striven to improve unhealthy diets and poor cooking habits in schools in the United Kingdom since 2005 when he launched his “Feed Me Better” campaign. Since 2006, junk food is banned in British schools, and new legal food-based standards for school food were brought in. Next to the UK, also Portugal has compulsory regulations on the provision of school lunches [22]. Several other European countries have also adopted measures concerning nutrition in schools. For instance, France has banned automatic vending machines and energy drinks in 2005 and 2008, respectively [22], and Spain has banned the sale in schools of food and drinks that have high amounts of saturated fat, trans fats, salt, or sugar in an effort to tackle a rising prevalence of overweight and obesity [23].

### 4.2. Practical Recommendations and Critical Remarks

Results indicate that support from a Municipal Health Service is highly appreciated. As mentioned before, Municipal Health Services do not always have enough time and manpower to assist all schools in need of support in their region. Therefore, it is to be recommended to keep the Canteen Brigade as an important component of the programme. The Brigade provides schools with tailored advice, so schools know where to start, which products are suitable for a healthy offering, and how to create a healthy school canteen. And indeed, the Brigade is increasingly being called upon by schools to help them change the cafeteria offerings. Another advantage of deploying a Brigade is that it reinforces the efforts of the Municipal Health Services, for example, by acting as interlocutor when talking with an extern caterer.

Another recommendation to ensure continuation of the programme that follows from the present findings is that schools should always be advised to include healthy nutrition in their school food policy. In addition, schools should strive for structural changes in the school cafeteria offerings and incorporate healthy eating in the regular curriculum. These factors should increase potential success. Also, schools can be informed about the “Healthy School Method” of the Centre for Healthy Living. This method promotes an integrated and structured approach to create a healthy school, including healthy cafeteria offerings.

A last recommendation concerns the Municipal Health services. To be better able to meet their need, it is advisable to allocate more time to them to recruit schools for the Stimulation Award competition.

Also, some critical remarks seem in place. First, it is noteworthy that, with respect to the near future, the atmosphere seems to be somewhat less favorable compared to five years ago. During the school year 2006-2007, 59% of the secondary schools expected to pay more attention to the issue of overweight in the near future, compared to 31% in 2011 [3]. In addition, a lower percentage of secondary schools (37% in 2011 compared to 46% during the school year 2006-2007) consider themselves to be coresponsible for the prevention of overweight among students [3]. Also worth mentioning is the fact that the offering of food and beverages appears to have become more healthy, but there was an increasing offer of pizza slices and sugary milk products [3]. This development could be a threat to achieving the goal of healthy school canteens in all Dutch schools in 2015.

In addition, some barriers concerning the content of the programme have to be tackled, as not all school directors report a shift from mostly unhealthy offerings before the programme to mostly healthy offerings currently. Unfortunately, we do not have full insight in completion of the programme: schools who participated in the Stimulation Award competition were included in the study. This implies they started with the programme, but sometimes it takes several months or even years to completely change the food on offer in the school canteen. As a result, it was not known whether the schools had successfully implemented the programme. Also, intractability of practice might play a role in this matter. There can always be crucial factors in some schools that negatively impact the process. From experience, we know most of the time these factors are lack of time or lack of support from other important parties within the school. Therefore, more has to be done to involve other important parties like students and parents. As a first step, The Netherlands Nutrition Centre has started using inspirational examples of other schools to motivate schools to organize activities for students, for instance, giving them an active role in composing and preparing the cafeteria offerings. This might actually work as a double-edged sword, as these actively engaged students might be perceived as role models by other students hereby creating a positive social norm that could encourage other students to pay more attention to their eating behaviour. Another matter that has room for improvement is information about time and finances that are needed for implementing the programme. Specifically, directors of nonparticipating schools indicate that time and finances are factors that play a role in deciding whether to participate or not. More information needs to be given about these factors so that schools can make well-informed decisions. In order to meet this need, a factsheet will be composed that provides directors with the information they need.

Also, schools have to be stimulated to only remove and not add unhealthy products. The School Canteen Brigade of The Netherlands Nutrition Centre will play an important role in this, educating schools how to execute to programme effectively and how to compose a healthy offering. Moreover, in the present study, it was found that school directors of schools with a caterer managed by the school itself perceived their cafeterias to have a healthier offering than schools directors of schools with an external caterer. This is a finding that needs attention. The Manifest on Healthier Food can play an important role in changing this situation, by committing different parties—including external caterers—to achieve a minimum of 75% healthy products according to the Dutch guidelines for healthy food.

### 4.3. Venues for Future Research

At this point, we would like to stress that, in the present descriptive study, only user perceptions have been measured and no quantitative data of food supplies in the school canteens of participating and nonparticipating schools were measured. Moreover, the programme's effect on students' actual eating behaviour has not been measured in this study. Whether the programme will prove to be effective in changing students' eating behaviour has yet to be demonstrated. However, previous research has established that changing aspects in the food environment can indeed affect eating behaviour. For example, aforementioned research in The Netherlands by TNO has clearly demonstrated that making offerings healthier can change students' choices in a positive way and that changes in assortment can lead to changes in consumption [15]. In addition, evaluation research conducted in the US of a school-based intervention on replacing food items with low nutritional value with more healthy ones has demonstrated that this was an effective means of decreasing students' consumption of unhealthy food [24]. Moreover, there was no evidence for a so-called compensation effect: students did not engage in compensatory consumption of unhealthy food at home. Also, the present programme bears resemblance to theories about “nudging” that posit that people can be gently “pushed” toward healthier life choices by making minor adjustments in their environment or choice architecture [25]. The essence of the nudging approach is to elegantly use common decision heuristics that ordinarily steer people toward unhealthy decisions instead to nudge them in a healthier or more beneficial direction [26]. For example, research has shown that making unhealthy options less accessible helps people to choose healthier options [26]. Together, these two lines of research provide indirect evidence that the present approach of creating a healthier offering in school cafeterias could actually result in healthier food choices and eating behaviour. However, further research is necessary to determine whether such a positive effect actually occurs.

More research is also needed to get more insight in effects and barriers that might operate in each of the four different steps of the programme. The current research has not taken this into account. In addition, it might be interesting to examine whether there are differences between participating schools and nonparticipating schools in domains like health issues and neighbourhood socioeconomic status. Future research should give more insight in these differences as this might provide new starting points to recruiting and stimulate schools to enroll in the programme.

## 5. Conclusions

The environment can exert a strong influence on people's food decisions. In order to facilitate students to make more healthy food choices and to develop healthy eating habits, it is therefore important that the school food environment is healthy. The Healthy School Canteen programme is an intervention that helps schools to make their cafeteria's offering healthier. The present study shows that this intervention is promising, as directors and students of participating schools perceive their cafeteria's offering to be healthier after implementing the programme than prior to implementation, and participating schools perceive their cafeteria's offering to be healthier than nonparticipating schools.

It will be a challenge to motivate schools to enroll in the programme in order to achieve the goal of all school cafeterias in The Netherlands being healthy by 2015. While it is promising that one-third of schools are (or have been) participating in the programme, still two-thirds of schools have not participated yet. And as a lower percentage of secondary schools consider themselves to be coresponsible for the prevention of overweight among their students, it may be necessary and fruitful to explore other ways to attain this goal. Possibly, we could take France and Spain as an example and use legislation to create healthy school cafeterias in every school in The Netherlands.

With the present obesity epidemic and the number of young people in The Netherlands being overweight continuing to grow, any measures that may help in facilitating healthy food choices deserve to be put into consideration. In light of the Dutch government's preference for stimulating individuals to make their own health decisions, interventions that are aimed at creating a healthier environment that enables and facilitates people to make healthy decisions are a particularly fruitful venue to be further explored.
